# Co-Circulation of Bovine Leukemia Virus Haplotypes among Humans, Animals, and Food Products: New Insights of Its Zoonotic Potential

**DOI:** 10.3390/ijerph18094883

**Published:** 2021-05-04

**Authors:** Adriana P. Corredor-Figueroa, Nury N. Olaya-Galán, Sebastian Velandia-Álvarez, Marina Muñoz, Sandra P. Salas-Cárdenas, Milcíades Ibáñez-Pinilla, Manuel A. Patarroyo, Maria F. Gutiérrez

**Affiliations:** 1 Grupo de Enfermedades Infecciosas, Laboratorio de Virología, Departamento de Microbiología, Pontificia Universidad Javeriana, Bogota 110231, Colombia; acorredorf@ecci.edu.co (A.P.C.-F.); velandia.s@javeriana.edu.co (S.V.-Á.); sandra.s909@gmail.com (S.P.S.-C.); mfgutier@javeriana.edu.co (M.F.G.); 2 Vicerrectoría de Investigación, Universidad ECCI, Bogota 111311, Colombia; 3 PhD Program in Biomedical and Biological Sciences, Universidad del Rosario, Bogota 111221, Colombia; 4 Centro de Investigaciones en Microbiología y Biotecnología-UR (CIMBIUR), Facultad de Ciencias Naturales, Universidad del Rosario, Bogota 111221, Colombia; claudia.munoz@urosario.edu.co; 5 Research Department, Hospital Universitario Mayor Méderi—Universidad del Rosario, Bogota 111411, Colombia; milciades.ibanez@urosario.edu.co; 6 Molecular Biology and Immunology Department, Fundación Instituto de Inmunología de Colombia (FIDIC), Bogota 111321, Colombia; mapatarr.fidic@gmail.com; 7 Microbiology Department, Faculty of Medicine, Universidad Nacional de Colombia, Bogota 111321, Colombia; 8 Health Sciences Division, Main Campus, Universidad Santo Tomás, Bogota 110231, Colombia

**Keywords:** molecular epidemiology, bovine leukemia virus, haplotypes, zoonoses, recombination analysis

## Abstract

Bovine leukemia virus (BLV) is the causative agent of leukemia/lymphoma in cattle. It has been found in humans and cattle-derived food products. In humans, it is described as a potential risk factor for breast cancer development. However, the transmission path remains unclear. Here, a molecular epidemiology analysis was performed to identify signatures of genetic flux of BLV among humans, animals, and food products. Sequences obtained from these sources in Colombia were used (*n* = 183) and compared with reference sequences available in GenBank. Phylogenetic reconstruction was performed in IQ-TREE software with the maximum likelihood algorithm. Haplotype (hap) distribution among the population was carried out with a median-joining model in Network5.0. Recombination events were inferred using SplitsTree4 software. In the phylogenetic analysis, no specific branches were identified for the Colombian sequences or for the different sources. A total of 31 haps were found, with Hap 1, 4, 5 and 7 being shared among the three sources of the study. Reticulation events among the different sources were also detected during the recombination analysis. These results show new insights about the zoonotic potential of BLV, showing evidence of genetic flux between cattle and humans. Prevention and control strategies should be considered to avoid viral dissemination as part of the One Health program policies.

## 1. Introduction

Zoonoses are described by the World Health Organization and the Centers for Disease Control and Prevention as any disease or infection transmitted naturally within the human–animal interface (e.g., direct contact with animals and its environment, animals’ body fluids, animal-derived food products), leading to the emergence of new or previously unknown infections in humans [1,2]. Zoonotic infections have a special impact in developing countries, due to a higher exposure to risk factors such as contact with animal sources, low hygiene conditions and contamination of water sources [3,4]. One of the major threats for these infections is through the consumption of unindustrialized, undercooked, and raw food products mainly obtained from livestock or poultry, which could be infected or contaminated with pathogens [5]. Determining the complete pathway of zoonotic infections is challenging due to the several steps that are needed for spillover of infections and epidemiological detection of specific outbreaks as well as the identification and characterization of circulating pathogens [6].

Bovine Leukaemia Virus (BLV) is a retrovirus within the *deltaretrovirus* genus; is included within the five most important viral agents in livestock production and is the causative agent of enzootic bovine leukosis, which is a lymphoproliferative chronic disease which mainly affects cattle herds. Most of the infected cattle (70%) remain without any clinical signs, while some of them evolve into leukemia and/or lymphoma (10–15%) after a persistent infection over several years [7]. BLV is distributed worldwide with prevalence rates between 5 and 90% among different regions [8], with a particular impact both in North and Latin American countries with the highest reported prevalence rates [9,10,11,12].

Besides cattle, some other animal species are also susceptible to infection by BLV, including buffaloes, sheep, goats and alpacas [13,14,15,16,17]. BLV has been proposed in the literature as a pathogen with the capability to infect multiple species. This has been shown both naturally and experimentally, demonstrated by BLV’s capacity to induce leukemia/lymphoma in experimental animals and its capacity to infect multiple cell lines obtained from different sources [18,19,20]. Thus, the hypothesis of trespassing the species-specific barrier in natural environments has been proposed, supported by the broad range of mammals that BLV can infect, possibly through shared receptors between species [21,22]. The presence of the virus in different species as part of the natural course of BLV infection makes it difficult to implement and execute prevention and control strategies in livestock production, favoring transmission processes in mixed herds (e.g., sheep, goats, and cattle).

In addition, evidence of BLV in human beings has been reported by different researchers via the presence of its gene segments, proteins and antibodies against the virus in different parts of the world [23,24,25,26,27,28]. Epidemiological studies have identified a significant association between the presence of the virus and breast cancer development, proposing BLV as an intermediate risk factor for cancer outcome [29,30]. Furthermore, evidence of BLV and some other viruses has also been reported in lung cancer, showing a correlation with the up and down regulation of metabolic pathways associated with cell control and oncogenesis [31,32]. Even though the presence of the virus in humans has been reported, further studies are needed to completely understand its mechanisms related with oncogenesis as well as its transmission pathways in this specific population. Hence, there are still a considerable knowledge gaps as regards the interactions of animal species and human beings.

In cattle, the transmission pathways are well characterized, involving: processes of direct contact between body fluids; transmission through iatrogenic procedures, such as vaccination; dehorning and insemination processes [7]. Moreover, vertical transmission by breastfeeding from cows to calves has also been reported. Nonetheless, these transmission pathways between species and into humans are not clear, but it has been hypothesized that transmission to humans occurs through the consumption of animal-food products that might be infected with the virus [27].

Previous studies by our research group have found the presence of the virus both in cattle [10] and humans [33], as well as the presence of viral gene segments in raw beef and milk [34]. However, no concluding remarks have been described towards the proposal of a zoonotic infection from cattle to humans, hence, the transmission pathway has not been clarified. In order to advance the knowledge of BLV’s transmission patterns, this study was aimed at understanding the transmission profiles of the virus when it is present in different sources (cattle, humans, and food products), through a comprehensive phylogenetic analysis between sequences obtained from the above-mentioned sources as well as by identifying recombination events between viral isolates, and risk factors of exposure in the human population.

## 2. Materials and Methods

### 2.1. Population and Samples

Samples stored at −20 °C at the Virology Lab of the Pontificia Universidad Javeriana obtained between 2015–2018 and collected from cattle blood [10], human female breast tissues and blood [33] and cattle-derived food products (milk and beef) [34] from different regions of Colombia were used in this study. In these samples, presence of BLV had previously been detected, positive samples were sequenced and deposited in GenBank.

Briefly, total DNA extraction was performed using High Pure PCR Template Preparation Kit (Roche Applied Science^®^, Mannheim, Germany) following the manufacturer’s instructions. BLV detection was carried out via PCR amplification, with the PCR Master (Roche Applied Science^®^, Mannheim, Germany) targeting the *gag* region of the virus (nt 1068–1453; PF–AACACTACGACTTGCAATCC; PR–GGTTCCTTAGGACTCCGTCG). To increase the sensitivity of the PCR, in some cases (mainly female samples and food products), a nested PCR was performed (nt 1097–1369) [27]. Subsequently, positive samples of BLV for this region were sequenced using Sanger technology in Macrogen Inc. (Seoul, Korea) using the same two primers used to detect the viral presence and the final sequences were deposited in the GenBank repository for further analyses.

### 2.2. Data Retrieval and Viral Target Region

For the molecular phylogenetic analysis, Colombian sequences from the three sources were downloaded from GenBank (cattle—MH293473.1 to MH293501.1, humans—MN831896 to MN831962, and food products—MH057402.1 to MH057465.1) as well as some other reference sequences. A total of 259 sequences were included in the analysis. Of these, 64 of them were obtained from the peripheral blood of cattle, 67 were obtained from breast tissue and the blood of Colombian females with and without breast cancer; 29 were from cattle-derived food products; 27 were obtained from women breast tissue; finally, 72 reference sequences of the complete BLV genome—obtained from cattle around the world—were used. Complete BLV genome sequences available in GenBank were used as the reference sequences. Supplementary Tables S1 and S2 show the details of the sequences included in the study.

Initially, Colombian sequences were aligned using MAFFT (v7.427—2019, available at https://mafft.cbrc.jp/alignment/software/ (accessed on 27 March 2021), Kyoto, Japan), under the automatic settings of the program [35,36]. After alignment, a diversity analysis of the Colombian sequences was carried out in DNAsp [37] to select the dataset with the most informative region within the *gag* gene.

A region of 182 base pairs was selected for further analyses, considering the region with the greatest number of haplotypes within the analyzed population and the least number of loses due to the quality of Sanger sequencing in the extremes of the sequences. Later, the same frameshift was filtered in the reference sequences of BLV sequences obtained from GenBank.

### 2.3. Phylogenetic Analyses

After multiple alignment of the complete data set was obtained, a phylogenetic reconstruction was conducted in IQ-TREE software multicore version 1.6.12 [38]. A previous selection of the most relevant nucleotide substitution model in ModelFinder [39] was carried out with 1000 ultrafast bootstrap replicates using UFBoot2 [40]. Branching support metrics with aLRT [41] and its nonparametric equivalent SH-aLRT [42], with 1000 replicates, were also considered. The final edition of the phylogenetic tree was carried out in the ITOL program available online (https://itol.embl.de (accessed on 25 June 2020)). 

### 2.4. Haplotype Distribution and Network Analysis

The haplotypes in the evaluated dataset were identified with a Fasta matrix, constructed for haplotype network analysis in the DNA alignment software (Fluxus Technology Ltd., Colchester, UK, available at http://www.fluxus-engineering.com/align.htm (accessed on 27 March 2021)). In parallel, the median-joining model, based on 1000 iterations with default parameters in Network 5.0 software, was used (Fluxus Technology Ltd., Colchester, England, available at http://www.fluxus-engineering.com/sharenet.htm (accessed on 27 March 2021), [43,44]).

### 2.5. Identification of Recombination Events between Colombian Isolates Obtained from Cattle, Food Products and Humans

After haplotypes in the data set were identified, recombination analyses were performed to identify the molecular rearrangements among the sequences, represented by reticulation events among different sources. For this purpose, a representative sequence for each haplotype was selected and the consequent haplotype alignment was used as the input for the phylogenetic networks. Analyses were performed using the Neighbor-Net method [45], available in the SplitsTree4 package (Version 4.14-4, Tübingen, Germany) with 1000 iterations. Finally, recombination indexes were determined with DNAsp (v.5.0, Barcelona, Spain) as markers of genetic diversity in the analyzed population based on the haplotypes detected.

### 2.6. Identification of Risks of Exposure to BLV in Humans and Association with Circulating Haplotypes

Human samples were taken from a cohort of patients between 2016 and 2018 with benign and malignant breast tumors, being treated at the breast surgical service at Méderi Hospital (MH) located in Bogotá, Colombia. In addition, samples were also collected from a secondary population of deceased females without tumor development on the breast.

The study was approved by the ethics committee of Universidad del Rosario (UR) and Méderi Hospital (Record No. CEI-ABN026-000 241, 2016). All procedures were performed in accordance with the ethical standards of the institution and with the 1964 Helsinki declaration and its later amendments (last revision 2013). All the participants or relatives (in the cases of deceased females) voluntarily signed an informed consent form prior to sample collection. Data obtained during the study was used under confidentiality.

After the informed consent was signed, a survey of the participants/relatives was conducted to collect information regarding possible exposure factors related with the acquisition of BLV from cattle (i.e., direct contact, living in shared environments, contact with blood of cattle, and consumption of cattle-derived food products including meat, dairy products, and raw milk). As for deceased females for whom the quality of information was not accurate and incomplete, missing data were excluded from the analysis. A risk assessment was performed for patients from Mederi Hospital, for whom an association between the presence of the virus and breast cancer had previously been identified [33].

Chi-square bivariate analysis was performed to identify if any of the above-mentioned factors were significant for the presence of BLV in humans. Afterwards, a Mann–Whitney analysis was performed to detect the effect of multiple variables in the model, followed by a multinomial logistic regression to identify Odd Ratios (OR) values related with the acquisition of the virus. Finally, a chi-square bivariate analysis was performed between the obtained haplotypes in humans and the exposure factors obtained in the study. Results were considered statistically significant if they had a *p* value of <0.05 with a confidence interval of 95%. Statistical analyses were performed in SPSS (Ver. 25.0, IBM Corp., Amonk, NY, USA) and STATA (Ver. 15, StataCorp LP, College Station, TX, USA).

## 3. Results

### 3.1. Phylogenetic Analyses

**Figure 1:**
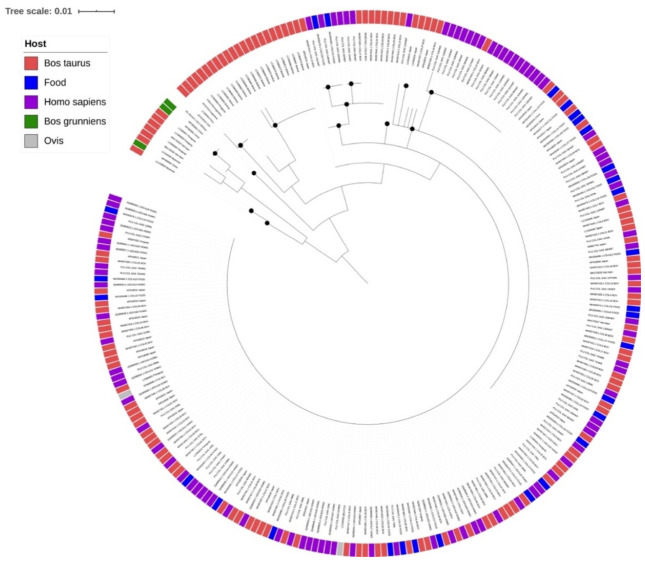
IQ-TREE phylogenetic reconstruction obtained from the multiple alignment performed in MAFFT of the complete data set (GenBank reference sequences and Colombian sequences). A region of 182 pb of the *gag* gene is shown. Colors indicate source of the virus. Red—bovine, Blue—food, Purple—humans. Jukes–Cantor substitution model was used in IQ-TREE. Black dots represent nodes higher than 90%, with a bootstrap of 1000 replicates.

The comprehensive phylogenetic analysis showed common characteristics among the Colombian sequences of BLV obtained from cattle, food, and humans. In the phylogenetic reconstruction with IQ-TREE, a Jukes–Cantor model was identified as the best substitution model (Figure 1). It was found that the virus obtained from different sources had a heterogeneous distribution, with no specific branches within the human, cattle, or food sequences. When compared with the sequences available in GenBank, mixed patterns were found among the Colombian human, food, and bovine sequences. From this, it can be inferred that the virus is circulating among the analyzed sources and does not generate specific clusters among the sequences of the data set due to its heterogeneous distribution.

### 3.2. Haplotype Distribution and Network Analysis

**Figure 2:**
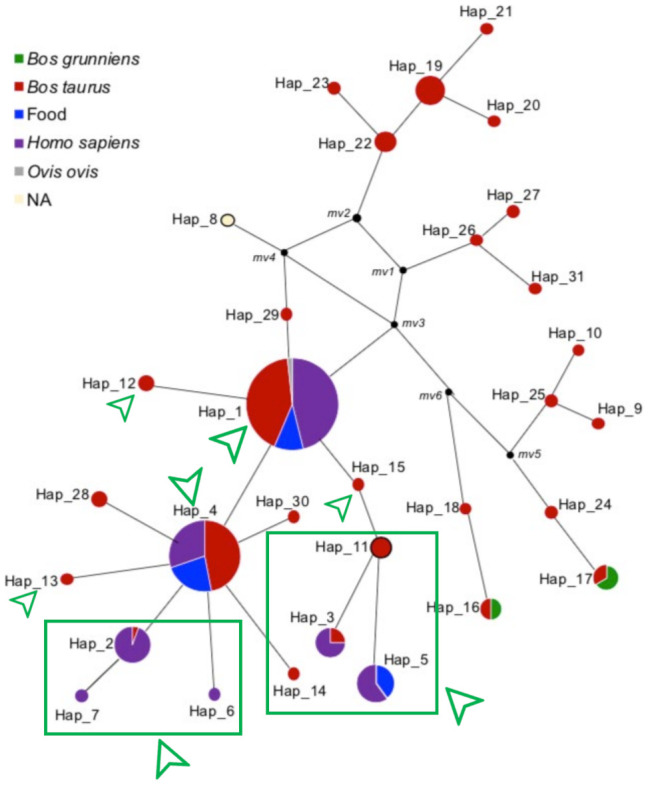
Haplotype network of Bovine Leukaemia Virus (BLV) obtained from the multiple alignment of the complete data set, performed with Median-Joining tool in Network v.5.0. A total of 31 haplotypes were identified in the analyzed dataset. The size of the circles represents the frequency of the haplotypes in the study. Hap 1 (*n* = 117) and Hap 4 (*n* = 66) were the most frequent haplotypes. Distances from haplotypes represent the sequence-type differences as regards nucleotide substitutions in the data set Colors indicate source of the virus. Red—bovine, Blue—food, Purple—humans. Notice the three colors in Hap 1 and 4. Small black circles were generated automatically to estimate connectors of the analyzed sequences. Haplotypes shown in green squares and arrows indicate those in which Colombian sequences were identified.

A haplotype network analysis (Figure 2) was carried out to evaluate the distribution patterns of BLV among the three populations, potential dissemination profiles and the eventual transmission networks between cattle and humans. Thirty-one haplotypes were identified (Hap 1–31) in the complete data set with a haplotype diversity of 0.7256. The most predominant haplotype was Hap 1 (*n* = 117) followed by Hap 4 (*n* = 66).

In the haplotype network, the Colombian sequences obtained from the three evaluated sources were mainly distributed in Hap1 and Hap4 (identified with green squares and arrows, Figure 2). Nucleotide changes among Hap 1 and 4 were shared between cattle, food, and humans. In Hap 1, sequences from Japan, Uruguay, and Paraguay were also found. In Hap 4, sequences from other countries including Japan, China and Vietnam were also found.

Haps 1 and 4 showed other variations such as those found in Haps 2, 7 and 6 for humans and Haps 19 to 23 in bovines. These haplotypes were more divergent than those shared among all the sources evaluated. Here, Haps 2, 3, 5–7, and 11–14 were exclusive to the sequences obtained from sources in Colombia. Shared haplotypes between sources of the virus were observed in Haps 2, 3 and 5, although no evidence of sequences from other countries was identified.

In the other haplotypes identified in the study, no sequences from Colombia were found but certain specific clusters by countries were visualized, as in the case of the Bolivian sequences in Hap 19. In Haps 28 and 30, which have a phylogenetic closeness to the Colombian nodes, sequences obtained from Japanese cattle were identified.

### 3.3. Identification of Recombination Events

**Figure 3:**
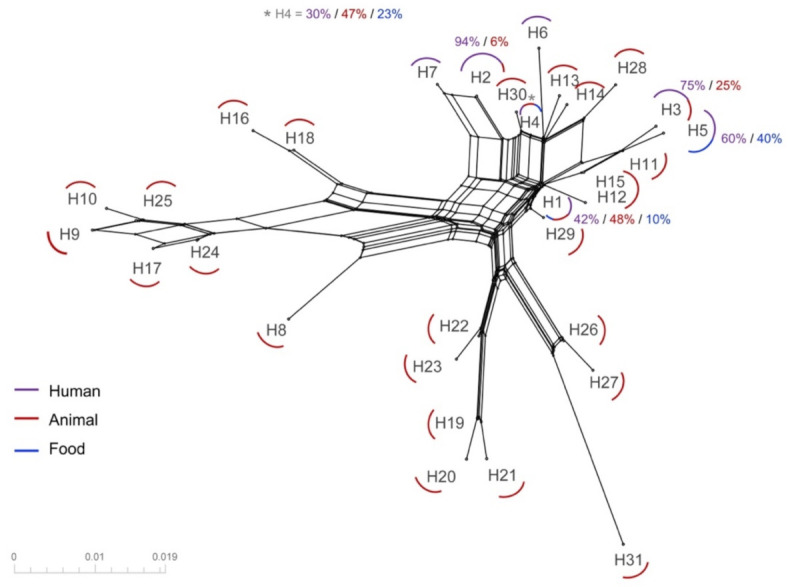
Phylogenetic network obtained from the alignment of representative haplotypes based on Neighbor-Net algorithm performed on SplitsTree. Reticulation events among haplotypes were identified, including both Colombian sequences and those from other regions. Colors represent sample source: Red—bovine, Blue—food, Purple—humans. Reticulation events between mixed sources were identified, particularly for Haps 1 and 4 at the central network hub, in which the three sources of the virus were identified.

The recombination analyses performed on the complete dataset in the SplitTree program (Figure 3) revealed reticulation events between the identified haplotypes, representing evidence of recombination signals. These reticulation events were identified at the intersections of the network, in which sequences from cattle, humans and food are shared, evidencing the flow of genetic information between cattle and humans.

Recombination events were mainly identified by crosslinking in the middle of the network, led by Hap 1 and Hap 4, and with the absence of specific branches for sequences of human origin. Several recombination signals were observed in the sequences, indicating that the fragment used has sufficient resolution power (genetic divergence markers) to compare the virus among sources.

A total of 36 polymorphic sites were identified, with a minimum of five recombination events determined in DNasp. The genetic diversity index showed a variance of the distribution of the samples (Sk ^ 2) of 9213, with a θ index of 2671 per gene (R < 0.0001) between adjacent sites. It should be noted that with these results, recombination events were found in the exclusive bovine haplotypes, which are associated with the genetic diversity in its natural host. Likewise, a flow of genetic diversity transmitted to humans was found, shared by reticulation events among the three sources.

Haps 1 to 7, which were found in humans, were also found in sequences obtained from bovines, three of which (Haps 1, 4 and 5) were from food products. In addition, the haplotypes with co-circulation among the three evaluated sample sources were associated with the most reticulation events, providing evidence of genetic exchange among haplotypes found in livestock, food, and humans, as a sample of the flow of genetic information between sources.

### 3.4. Risks of Exposure to BLV and Haplotypes Association

BLV was previously detected by our research group in women with different diagnoses (breast cancer, benign pathology of the breast, no tumor development), and a significant association was identified between the presence of the virus and breast cancer in the Colombian female population [33]. Here, with the same basis population (*n* = 168) for cancer risk assessment, in which BLV was present in 61.3% of malignant samples and 46.5% of benign samples, we analyzed the potential exposure factors related to acquiring the virus independently of the diagnoses obtained in the participants. Deceased females (participants with no tumor development) were excluded from the risk analysis due to missing data retrieved from the relatives.

In the bivariate analysis, a correlation between the consumption of dairy products such as home-made natural yoghurt and flavored yoghurt as well as raw milk and number of dairy products were significant for the presence of the virus (*p* < 0.05). Age (≥50, <50) and city of origin of the participants (capital city or other) also showed significance for the analysis. Meat consumption, direct contact with cattle and body fluids, and living in shared environments with cattle were not significant for the study. Sociodemographic characteristics and risk of exposure regarding the presence of BLV are shown in Table 1. In the multinomial logistic regression, it was shown that females who had a higher consumption of dairy products, also had a higher risk of acquisition of BLV (OR = 2.424, CI 95%: 1.063–5.527, *p* = 0.035, Table 2).

Analysis of the correlation of circulating haplotypes with the exposure factors in the human population did not show statistical differences (*p* ≥ 0.05). Haplotypes of BLV were evenly distributed among female samples with different exposure factors. In females with consumption of milk, dairy products, and beef, were present all the haplotypes of BLV reported in the study. The frequencies of occurrences of haplotypes and exposure factors are shown on Supplementary Table S3.

## 4. Discussion

Thus far, the studies that have been carried out since the molecular characterization of the BLV have been focused, above all, on genotyping and characterizing the worldwide viral distribution, emphasizing its molecular epidemiology [8]. However, few studies have focused on the diversity of the virus, nor on the dispersion and circulation patterns that can occur in the natural course of infection, considering both cattle as its natural host, and the interaction with the environment and arrival at accidental hosts, such as sheep and humans. In the current study, BLV sequences obtained from cattle, food, and humans were analyzed with the purpose of understanding the flux and transmission dynamics that the virus might possess to reach humans.

Shared distributions of the virus were identified among these three sources of analysis, indicating that the presence of the virus in humans is not an isolated event. Indeed, on the contrary, the results showed the co-circulation of haplotypes among the different sources, exhibiting recombination signatures, indicating that a common origin is shared. For the first time, these findings demonstrate genetic exchange among heterogenous hosts in the molecular marker used. Hence, this supports the hypothesis of the existence of transmission routes in the same ecological niche between cattle and humans.

Contemplating the globality of this study, there is evidence of congruence between the analyses carried out, revealing the transmission networks of the virus between cattle and humans, potentially using food as a dissemination vehicle [34]. These findings support evidence provided by previous studies [27,29] suggesting the transmission of BLV to human beings based on the evidence of the virus in food products and the presence of the virus in the bovine population with high prevalence rates and its worldwide distribution [8,10]. Statistically significant results were also identified in the current study with the consumption of dairy products and raw milk in humans (Table 2), supporting the hypothesis of transmission through cattle-derived food products.

In terms of the zoonotic principles of transmission (direct or indirect contact with animals, their environment, derivates and body fluids) [3], it seems that—at least in the evaluated population—direct contact with cattle does not represent a high risk. Rather, risk is more related with cattle and milk distribution for human consumption. In addition, our results also showed a statistical significance with the age and city of origin of female participants (Table 2). This could be explained by longer exposure periods in older women during their lives to different risk factors related with the acquisition of the virus, increasing the probability of being infected. On the other hand, the origin of the participants could be related with accessibility to food products and a better quality (e.g., industrialized milk, meat of selected and controlled cattle) as, in the capital city (Bogotá), there is a higher level of control in slaughterhouses compared with smaller cities and towns in the surroundings of Bogota or even other regions in the country. Even if most of the participants were from Bogotá and lived there, the results showed a significant difference as regards the presence of the virus in people who lived in or were from other regions. However, it would be interesting to perform similar analyses in other regions of Colombia as well as in other countries in order to elucidate the impact of cattle-derived food products and their quality in terms of transmission of the virus to the human population. In our study, fewer people from other regions were included compared to those from Bogota, and thus, it was not possible to determine the impact of the virus in each specific region.

In the haplotype network (Figure 2), shared mutations in Haps 1 and 4 between sources of the virus indicate that these are closely related sequences, proposing the co-circulation of BLV haplotypes between bovines and humans. In addition, Haps 1 and 4 showed sequences from different countries that were obtained from cattle, which indicates that these changes in sequences are not specific to the Colombian population but are common profiles of the virus that are also present in other regions. These findings suggest that the presence of the virus in humans came from viruses circulating in cattle, through a common point of dissemination, such as cattle-derived food products.

Although the information obtained in this study is not enough to understand the origin of the virus in Colombia, it is interesting to evaluate the phylogenetic relationships that these sequences have in the dissemination flux (Figure 2). Likewise, it is important to highlight that the nodes located in the upper right part of the graph belong to sequences of cattle from different countries, indicating that these mutations already existed in reported sequences of the GenBank. On the contrary, on the left side of the graph, exclusive human haplotypes (Haps 6 and 7) were identified for the Colombian sequences, however, it does not refer to a specific evolutionary profile in humans but probably this suggests missing data at the population level, restricting detailed monitoring of the flow of the virus from cattle to humans. 

Considering that this is the first study aimed at comparing sequences obtained from humans, food, and cattle, it should be noted that no human sequences from other regions besides Colombia are available. The lack of these sequences does not allow one to obtain the complete panorama of the dissemination profiles of the virus. Additionally, it would be interesting to analyze these patterns among other populations and other genomic regions of the virus. It would be expected that haplotypes would be grouped per countries and sources, as in the case of Haps 1 and 4.

In the phylogenetic network (Figure 3), identification of reticulation events between the different sources is suggested. From these results, it could be inferred that the BLV present in cattle and humans contains recombination events that indicate the flux of genetic information among sources. Additionally, shared haplotypes between species indicate co-circulation of the virus between humans and cattle, as well as the presence of BLV in food products. From the reticulation events found in this study, a recombination process in cattle before arriving to human beings is expected to be occurring, as no evidence of human-to-human transmission has yet been reported for BLV. The results obtained in the current study could suggest that the virus presents a transmission pattern in heterogenous hosts.

In the study of diseases of zoonotic origin, examples of other viruses have been recorded in the literature. Rabies is a classic example of a zoonotic infection, although spillover into the human population is stopped [46]. Other cases, such as the current SARS-CoV-2 pandemic, demonstrate the rapid spread of diseases among the human population after the virus gains entrance into human beings [47,48]. Additionally, evidence of zoonotic infections through the consumption of animal-derived food products has been described, as in the case of hepatitis E, through the consumption of meat products obtained from pork [49,50]. Even though different models have been studied for emerging zoonotic infections from animals, the ability of infectious agents to remain in human beings differs among the different viral agents. This is mainly due to the capacity of the virus to perform complete and successful cycles in accidental hosts, as well as the impact on spillover among humans [51].

In the case of BLV, considering its worldwide distribution [8] and the high rates of consumption of beef and milk in human beings, it is possible that the virus can trespass the species barrier, thus, reaching humans. Our study demonstrated sequences with more than 95% of identity between cattle, humans and food products, no specific branches for each source in the phylogenetic analysis, and evidence of the most frequent haplotypes among the female participants. Haplotypes were equally distributed between the possible exposure factors for acquiring the virus in humans, and a significant correlation with the consumption of raw milk and dairy products was also found. Identification of these dynamics of transmission in BLV could guide the process of generating prevention and control strategies focused on its natural hosts, accidental hosts, reservoirs and ecological niches [3]. Even though further studies are still needed in order to elucidate the transmission factors for BLV infection to humans, prevention and control strategies should be considered to stop the viral spread worldwide, considering the One Health principle (humans, animals and environment as a whole) [52], as a result of the implications of the virus both in animal and human health.

## 5. Conclusions

In this study, it has been shown that BLV is circulating in humans, cattle, and food products in Colombia. Heterogenous distribution of the viral sequences was identified among sources, suggesting a transmission phase of the virus from bovines to humans based on the co-circulation of haplotypes among the evaluated sources. In addition, recombination signatures were detected in the phylogenetic networks. However, there is no evidence of human-to-human transmission, although the results obtained in this phylogenetic analysis suggest a genetic flux between the human–animal interface, probably through the consumption of infected food products.

## Figures and Tables

**Table 1:** Sociodemographic characteristics of female participants and exposure factors to BLV. Bi-variate analysis comparing the presence of the virus with the exposure factors.

	Viral Presence		
	Positive	Negative	*p* Value
	*n* (%)	*n* (%)	
Pathology diagnoses			
Malignant samples (*n* = 75)	46 (61.3)	29 (38.7)	<0.001
Benign samples (*n* = 85)	41 (48.8)	44 (51.2)	
Age			
≥50	39 (63.9)	22 (36.1)	0.039 *
<50	61 (49.6)	62 (50.4)	
Socio-demographic characteristics			
*Origin*			0.036 *
Bogotá	61 (49.3)	62 (50.4)	
Other	30 (66.7)	15 (33.3)	
*Educational level*			0.785
Elementary school	23 (52.3)	21 (47.7)	
High School	34 (58.6)	24 (41.4)	
Vocational and professional studies	34 (53.1)	30 (46.9)	
Risks of exposure to BLV			
Dairy Products consumption			
Flavored Yoghurt	70 (59.8)	47 (40.2)	0.023 *
Home-made natural yoghurt (Kumis)	61 (60.4)	40 (39.6)	0.042 *
Cheese	83 (55.0)	68 (45.0)	0.614
Jelly foot dessert (Gelatina de pata)	41 (60.3)	27 (39.7)	0.148
Industrialized milk	90 (54.2)	76 (45.8)	0.708
Direct contact with cattle	39 (55.7)	31 (44.3)	0.428
Amount of dairy products and raw milk			0.04 *
4 or more	31 (72.1)	12 (27.9)	
3	18 (52.9)	16 (47.1)	
2	4 (30.8)	9 (69.2)	
None	2 (66.7)	1 (33.3)	

* Significant results in the bivariate analysis for the viral presence were considered statistically significant for a *p* value <0.05.

**Table 2:** Multinomial logistic regression of the risks of exposure and viral presence in the human population. Odd-ratio values adjusted by age.

Variables	Viral Presence		
	β	OR (95% CI)	*p* Value
Age			
≥50	0.794	2.212 (1.111-4.402)	0.024
<50	--	1.00 (Reference)	--
City of origin			
Other	0.800	2.224 (1.030-4.805)	0.042
Bogota	--	1.00 (Reference)	--
Milk consumption and dairy products ^a^	--	--	0.037 **
Only milk or 1 dairy product	−0.990	0.372 (0.103-1.346)	0.132
Two dairy products/milk	0.000	1.00 (0.431-2.321)	1.000
Three or more dairy products/milk	0.885	2.424 (1.063-5.527) **	0.035 **
No consumption of dairy products	--	1.00 (Reference)	--

^a^ Amount of consumption of dairy products and raw milk. Dairy products include fresh cheese, flavored or natural yogurt, “gelatina de pata” (traditional dessert made with cattle collagen and milk). ** Significant results obtained for acquisition factors and the presence of the virus in the human population. *p* values <0.05 were considered statistically significant for the study.

## Data Availability

Data is contained within the article or supplementary material. The data presented in this study are available in GenBank (https://www.ncbi.nlm.nih.gov/genbank/) as described in the ‘Materials and Methods’ section and Supplementary materials (Tables S1 and S2).

## Supplementary Materials

The following are available online at https://www.mdpi.com/article/10.3390/ijerph18094883/s1, Table S1: Accession numbers and details of the sequences included in the BLV analysis–*gag* region. Table includes reference and own sequences. Table S2: Sequences obtained in the current study from women samples collected from breast and blood. Population description., Table S3: Haplotypes distribution among humans positive to BLV regarding their exposure factors for viral acquisition. Chi-square bivariate analysis of exposure factors vs haplotypes.

## References

[1] (2017). World Health Organization Zoonoses.

[2] CDC Zoonotic Diseases, One Health.

[3] Teshome H. (2019). Review on Principles of Zoonoses Prevention, Control and Eradication. Am. J. Biomed. Sci. Res..

[4] Bauerfeind R., von Graevenitz A., Kimmig P., Gerd-Schiefer H., Schwarz T., Slenczka W., Zahner H., Bauerfeind R., von Graevenitz A., Kimmig P., Gerd Schiefer H., Schwarz T., Slenczka W., Zahner H. (2016). Zoonoses: Infectious Diseases Transmissible from Animals to Humans.

[5] Karesh W.B., Dobson A., Lloyd-Smith J.O., Lubroth J., Dixon M.A., Bennett M., Aldrich S., Harrington T., Formenty P., Loh E.H. (2012). Ecology of zoonoses: Natural and unnatural histories. Lancet.

[6] Wang L.-F., Crameri G. (2014). Emerging zoonotic viral diseases. Rev. Sci. Technol. Off. Int. Epiz..

[7] Barez P.-Y., de Brogniez A., Carpentier A., Gazon H., Gillet N., Gutiérrez G., Hamaidia M., Jacques J.-R., Perike S., Neelature Sriramareddy S. (2015). Recent Advances in BLV Research. Viruses.

[8] Polat M., Takeshima S., Aida Y. (2017). Epidemiology and genetic diversity of bovine leukemia virus. Virol. J..

[9] LaDronka R.M., Ainsworth S., Wilkins M.J., Norby B., Byrem T.M., Bartlett P.C. (2018). Prevalence of Bovine Leukemia Virus Antibodies in US Dairy Cattle. Vet. Med. Int..

[10] Corredor-Figueroa A.P., Salas S., Olaya-Galán N.N., Quintero J.S., Fajardo Á., Soñora M., Moreno P., Cristina J., Sánchez A., Tobón J. (2020). Prevalence and molecular epidemiology of bovine leukemia virus in Colombian cattle. Infect. Genet. Evol..

[11] Polat M., Takeshima S.S., Hosomichi K., Kim J., Miyasaka T., Yamada K., Arainga M., Murakami T., Matsumoto Y., Barra Diaz V. (2016). A new genotype of bovine leukemia virus in South America identified by NGS-based whole genome sequencing and molecular evolutionary genetic analysis. Retrovirology.

[12] Virol A., Heinecke N., Tórtora J., Martínez H.A., González V.D., Hugo F., González-Fernández V.D., Ramírez H. (2017). Detection and genotyping of bovine leukemia virus in Mexican cattle. Arch. Virol..

[13] Selim A., Marawan M.A., Ali A.F., Manaa E., AbouelGhaut H.A. (2020). Seroprevalence of bovine leukemia virus in cattle, buffalo, and camel in Egypt. Trop. Anim. Health Prod..

[14] Feliziani F., Martucciello A., Iscaro C., Vecchio D., Petrini S., Grassi C., Bazzucchi M., De Carlo E. (2017). Bovine leukemia virus: Experimental infection in buffaloes and evaluation of diagnostic test reliability. Res. Vet. Sci..

[15] Lee L.C., Scarratt W.K., Buehring G.C., Saunders G.K. (2012). Bovine leukemia virus infection in a juvenile alpaca with multicentric lymphoma. Can. Vet. J. Rev. Vét. Can..

[16] Olson C., Kettmann R., Burny A., Kaja R. (1981). Goat lymphosarcoma from bovine leukemia virus. J. Natl. Cancer Inst..

[17] Nekoei S., Hafshejani T.T., Doosti A., Khamesipour F. (2015). Molecular detection of Bovine leukemia virus in peripheral blood of Iranian cattle, camel and sheep. Pol. J. Vet. Sci..

[18] Mammerickx M., Portetelle D., Burny A. (1981). Experimental Cross-Transmissions of Bovine Leukemia Virus (BLV) between Several Animal Species. Zent. Vet. R. B.

[19] Suzuki T., Ikeda H., Mase M. (2018). Restricted viral cDNA synthesis in cell lines that fail to support productive infection by bovine leukemia virus. Arch. Virol..

[20] Reichert M. (2017). Proteome analysis of sheep B lymphocytes in the course of bovine leukemia virus-induced leukemia. Exp. Biol. Med..

[21] Corredor A.P., Gonzales J., Baquero L.A., Curtidor H., Olaya-Galán N.N., Patarroyo M.A., Gutierrez M.F., González J., Baquero L.A., Curtidor H. (2018). In silico and in vitro analysis of boAP3d1 protein interaction with bovine leukaemia virus gp51. PLoS ONE.

[22] Bai L., Sato H., Kubo Y., Wada S., Aida Y. (2019). CAT1/SLC7A1 acts as a cellular receptor for bovine leukemia virus infection. FASEB J..

[23] Delarmelina E., Buzelin M.A., de Souza B.S., Souto F.M., Bicalho J.M., Falcão Câmara R.J., Resende C.F., Bueno B.L., Victor R.M., Florentino Galinari G.C. (2020). High positivity values for bovine leukemia virus in human breast cancer cases from Minas Gerais, Brazil. PLoS ONE.

[24] Mesa G., Ulloa J.C., Uribe A.M., Gutierrez M.F., Giovanna M., Carlos U.J., María U.A., Gutierrez M.F. (2013). Bovine Leukemia Virus Gene Segment Detected in Human Breast Tissue. Open J. Med. Microbiol..

[25] Khalilian M., Hosseini S.M., Madadgar O. (2019). Bovine leukemia virus detected in the breast tissue and blood of Iranian women. Microb. Pathog..

[26] Lendez P.A., Martinez-Cuesta L., Nieto Farias M.V., Shen H., Dolcini G.L., Buehring G.C., Ceriani M.C. (2018). Bovine leukemia virus presence in breast tissue of Argentinian women. Its association with cell proliferation and prognosis markers. Multidiscip. Cancer Investig..

[27] Buehring G.C., Shen H.M., Jensen H.M., Choi K.Y., Sun D., Nuovo G. (2014). Bovine Leukemia Virus DNA in Human Breast Tissue. Emerg. Infect. Dis..

[28] Buehring G.C., Philpott S.M., Choi K.Y. (2003). Humans have antibodies reactive with Bovine leukemia virus. AIDS Res. Hum. Retrovir..

[29] Buehring G.C., Sans H.M. (2020). Breast cancer gone viral? Review of possible role of bovine leukemia virus in breast cancer, and related opportunities for cancer prevention. Int. J. Environ. Res. Public Health.

[30] Khatami A., Pormohammad A., Farzi R., Saadati H., Mehrabi M., Kiani S.J., Ghorbani S. (2020). Bovine Leukemia virus (BLV) and risk of breast cancer: A systematic review and meta-analysis of case-control studies. Infect. Agents Cancer.

[31] Robinson L.A., Jaing C.J., Pierce Campbell C., Magliocco A., Xiong Y., Magliocco G., Thissen J.B., Antonia S. (2016). Molecular evidence of viral DNA in non-small cell lung cancer and non-neoplastic lung. Br. J. Cancer.

[32] Kim Y., Pierce C.M., Robinson L.A. (2018). Impact of viral presence in tumor on gene expression in non-small cell lung cancer. BMC Cancer.

[33] Olaya-Galán N.N., Salas-Cárdenas S.P., Corredor-Figueroa A.P., Rodriguez-Sarmiento J.L., Ibáñez-Pinilla M., Monroy R., Rubiano W., de la Peña J., Shen H., Buehring G.C. (2021). Evidence of bovine leukaemia virus in blood and breast tissues in Colombian women, a risk factor associated with breast cancer. J. Cancer Res. Clin. Oncol..

[34] Olaya-Galán N.N., Corredor-Figueroa A.P., Guzmán-Garzón T.C., Ríos-Hernandez K.S., Salas-Cárdenas S.P., Patarroyo M.A., Gutierrez M.F. (2017). Bovine leukaemia virus DNA in fresh milk and raw beef for human consumption. Epidemiol. Infect..

[35] Katoh K., Standley D.M. (2013). MAFFT multiple sequence alignment software version 7: Improvements in performance and usability. Mol. Biol. Evol..

[36] Katoh K., Misawa K., Kuma K.I., Miyata T. (2002). MAFFT: A novel method for rapid multiple sequence alignment based on fast Fourier transform. Nucleic Acids Res..

[37] Librado P., Rozas J. (2009). DnaSP v5: A software for comprehensive analysis of DNA polymorphism data. Bioinformatics.

[38] Nguyen L.T., Schmidt H.A., Von Haeseler A., Minh B.Q. (2015). IQ-TREE: A fast and effective stochastic algorithm for estimating maximum-likelihood phylogenies. Mol. Biol. Evol..

[39] Kalyaanamoorthy S., Minh B.Q., Wong T.K.F., Von Haeseler A., Jermiin L.S. (2017). ModelFinder: Fast model selection for accurate phylogenetic estimates. Nat. Methods.

[40] Hoang D.T., Chernomor O., von Haeseler A., Minh B.Q., Vinh L.S. (2018). UFBoot2: Improving the Ultrafast Bootstrap Approximation. Molecular biology and evolution. Mol. Biol. Evol..

[41] Anisimova M., Gascuel O. (2006). Approximate likelihood-ratio test for branches: A fast, accurate, and powerful alternative. Syst. Biol..

[42] Guindon S., Dufayard J.F., Lefort V., Anisimova M., Hordijk W., Gascuel O. (2010). New algorithms and methods to estimate maximum-likelihood phylogenies: Assessing the performance of PhyML 3.0. Syst. Biol..

[43] Huson D.H. (1998). SplitsTree: Analyzing and visualizing evolutionary data. Bioinformatics.

[44] Bandelt H.-J., Forster P., Röhl A. (1999). Median-Joining Networks for Inferring Intraspecific Phylogenies. Mol. Biol. Evol..

[45] Huson D.H., Bryant D. (2006). Application of phylogenetic networks in evolutionary studies. Mol. Biol. Evol..

[46] Fisher C.R., Streicker D.G., Schnell M.J. (2018). The spread and evolution of rabies virus: Conquering new frontiers. Nat. Rev. Microbiol..

[47] Zhu N., Zhang D., Wang W., Li X., Yang B., Song J., Zhao X., Huang B., Shi W., Lu R. (2020). A Novel Coronavirus from Patients with Pneumonia in China, 2019. N. Engl. J. Med..

[48] Ye Z.-W., Yuan S., Yuen K.-S., Fung S.-Y., Chan C.-P., Jin D.-Y. (2020). Zoonotic origins of human coronaviruses. Int. J. Biol. Sci..

[49] Szabo K., Trojnar E., Anheyer-Behmenburg H., Binder A., Schotte U., Ellerbroek L., Klein G., Johne R. (2015). Detection of hepatitis E virus RNA in raw sausages and liver sausages from retail in Germany using an optimized method. Int. J. Food Microbiol..

[50] Di Bartolo I., Angeloni G., Ponterio E., Ostanello F., Ruggeri F.M. (2015). Detection of hepatitis E virus in pork liver sausages. Int. J. Food Microbiol..

[51] Plowright R.K., Parrish C.R., McCallum H., Hudson P.J., Ko A.I., Graham A.L., Lloyd-Smith J.O. (2017). Pathways to zoonotic spillover. Nat. Rev. Microbiol..

[52] Degeling C., Johnson J., Kerridge I., Wilson A., Ward M., Stewart C., Gilbert G. (2015). Implementing a One Health approach to emerging infectious disease: Reflections on the socio-political, ethical and legal dimensions. BMC Public Health.

