# Supplementary material for: Co-Circulation of Bovine Leukemia Virus Haplotypes among Humans, Animals, and Food Products: New Insights of Its Zoonotic Potential
Source: Int J Environ Res Public Health. 2021 May 4;18(9):4883. doi: 10.3390/ijerph18094883 (PMC8124648; doi:10.3390/ijerph18094883)
Supplement: Supplementary file 1 [file ijerph-18-04883-s001.zip › ijerph-1143393-SI.pdf]

International Journal of
Environmental Research and Public Health

MDPI

Research Article

Co-circulation of Bovine Leukemia Virus haplotypes among humans, animals, and food products: new insights of its zoonotic potential

Adriana P. Corredor-Figueroa [1,2,*,†], Nury N. Olaya-Galán [1,3,*,†], Sebastian Velandia-Álvarez [1], Marina Muñoz [4], Sandra P. Salas-Cárdenas [1], Milcíades Ibáñez-Pinilla [5], Manuel A. Patarroyo [6,7,8], Maria F. Gutiérrez [1]

Supplementary material

Table S1. Accession numbers and details of the sequences included in the BLV analysis – *gag* region. Table includes reference and own sequences.

Accession Number	Country	Host	Isolation Source	Hap.
AB987702	Japan	Bos taurus	Lymph	4
AF033818	USA	NA	Clone	8
AF257515	Argentina	Bos taurus	Blood	31
AP018006	Japan	Bos taurus	Blood	4
AP018007	Japan	Bos taurus	Blood	1
AP018008	Japan	Bos taurus	Blood	1
AP018010	Japan	Bos taurus	Blood	4
AP018011	Japan	Bos taurus	Blood	1
AP018012	Japan	Bos taurus	Blood	1
AP018013	Japan	Bos taurus	Blood	1
AP018014	Japan	Bos taurus	Blood	4
AP018015	Japan	Bos taurus	Blood	1
AP018016	Japan	Bos taurus	Blood	1
AP018017	Japan	Bos taurus	Blood	4
AP018018	Japan	Bos taurus	Blood	1
AP018019	Japan	Bos taurus	Blood	30
AP018020	Japan	Bos taurus	Blood	29
AP018021	Japan	Bos taurus	Blood	1
AP018023	Japan	Bos taurus	Blood	1
AP018024	Japan	Bos taurus	Blood	4
AP018025	Japan	Bos taurus	Blood	4
AP018026	Japan	Bos taurus	Blood	4
AP018028	Japan	Bos taurus	Blood	4
AP018029	Japan	Bos taurus	Blood	1

Accession Number	Country	Host	Isolation Source	Hap.
AP018030	Japan	Bos taurus	Blood	1
AP018031	Japan	Bos taurus	Blood	1
AP018032	Japan	Bos taurus	Blood	1
EF600696	FLK	Ovis ovis	Cell line	1
FJ914764	Argentina	Bos taurus	Blood	26
HE967302	Uruguay	Bos taurus	Lymphosarcoma	1
HE967303	Uruguay	Bos taurus	Lymphosarcoma	1
JQ480623.1	Colombia	Homo Sapiens	Breast	1
JQ480624.1	Colombia	Homo Sapiens	Breast	1
JQ480625.1	Colombia	Homo Sapiens	Breast	1
JQ480626.1	Colombia	Homo Sapiens	Breast	1
JQ480627.1	Colombia	Homo Sapiens	Breast	1
JQ480628.1	Colombia	Homo Sapiens	Breast	1
JQ480629.1	Colombia	Homo Sapiens	Breast	1
JQ480630.1	Colombia	Homo Sapiens	Breast	1
JQ480631.1	Colombia	Homo Sapiens	Breast	1
JQ480632.1	Colombia	Homo Sapiens	Breast	1
JQ480633.1	Colombia	Homo Sapiens	Breast	1
JQ480634.1	Colombia	Homo Sapiens	Breast	1
JQ480635.1	Colombia	Homo Sapiens	Breast	1
JQ480636.1	Colombia	Homo Sapiens	Breast	1
JQ480637.1	Colombia	Homo Sapiens	Breast	1
JQ480638.1	Colombia	Homo Sapiens	Breast	1
JQ480639.1	Colombia	Homo Sapiens	Breast	1
JQ480640.1	Colombia	Homo Sapiens	Breast	1
JQ480641.1	Colombia	Homo Sapiens	Breast	1
JQ480642.1	Colombia	Homo Sapiens	Breast	1
JQ480643.1	Colombia	Homo Sapiens	Breast	1
JQ480644.1	Colombia	Homo Sapiens	Breast	1
JQ480645.1	Colombia	Homo Sapiens	Breast	1
JQ480646.1	Colombia	Homo Sapiens	Breast	1

Accession Number	Country	Host	Isolation Source	Hap.
JQ480647.1	Colombia	Homo Sapiens	Breast	1
JQ480648.1	Colombia	Homo Sapiens	Breast	1
JQ480649.1	Colombia	Homo Sapiens	Breast	1
LC005615	Japan	Bos taurus	Lymphosarcoma	28
LC005616	Japan	Bos taurus	Lymphosarcoma	28
LC080651	Paraguay	Bos taurus	Blood	1
LC080652	Paraguay	Bos taurus	Blood	1
LC080653	Paraguay	Bos taurus	Blood	1
LC080654	Peru	Bos taurus	Blood	27
LC080655	Paraguay	Bos taurus	Blood	26
LC080656	Paraguay	Bos taurus	Blood	25
LC080657	Paraguay	Bos taurus	Blood	17
LC080658	Paraguay	Bos taurus	Blood	24
LC080659	Bolivia	Bos taurus	Blood	22
LC080660	Bolivia	Bos taurus	Blood	23
LC080661	Bolivia	Bos taurus	Blood	22
LC080662	Bolivia	Bos taurus	Blood	22
LC080663	Bolivia	Bos taurus	Blood	22
LC080664	Bolivia	Bos taurus	Blood	19
LC080665	Bolivia	Bos taurus	Blood	21
LC080666	Bolivia	Bos taurus	Blood	19
LC080667	Bolivia	Bos taurus	Blood	19
LC080668	Bolivia	Bos taurus	Blood	19
LC080669	Bolivia	Bos taurus	Blood	20
LC080670	Bolivia	Bos taurus	Blood	19
LC080671	Bolivia	Bos taurus	Blood	19
LC080672	Bolivia	Bos taurus	Blood	19
LC080673	Bolivia	Bos taurus	Blood	19
LC080674	Bolivia	Bos taurus	Blood	19
LC080675	Bolivia	Bos taurus	Blood	19
LC154848	Myanmar	Bos taurus	Blood	18
LC154849	Myanmar	Bos taurus	Blood	16
LC164083	pBLV-FLK	Ovis ovis	Blood	1
LC164085	Japan	Bos taurus	Blood	4
LC164086	Japan	Bos taurus	Blood	4
MF580991	China	Bos grunniens	Blood	17
MF580992	China	Bos grunniens	Blood	17
MF580995	China	Bos grunniens	Blood	16
MG800834	China	Bos taurus	Blood	4

Accession Number	Country	Host	Isolation Source	Hap.
MH057402.1	Colombia	Bos taurus	Blood	11
MH057403.1	Colombia	Bos taurus	Blood	1
MH057404.1	Colombia	Bos taurus	Blood	1
MH057405.1	Colombia	Bos taurus	Blood	4
MH057406.1	Colombia	Bos taurus	Blood	1
MH057407.1	Colombia	Bos taurus	Blood	4
MH057408.1	Colombia	Bos taurus	Blood	4
MH057409.1	Colombia	Bos taurus	Blood	4
MH057410.1	Colombia	Bos taurus	Blood	4
MH057411.1	Colombia	Bos taurus	Blood	4
MH057412.1	Colombia	Bos taurus	Blood	4
MH057413.1	Colombia	Bos taurus	Blood	1
MH057414.1	Colombia	Bos taurus	Blood	15
MH057415.1	Colombia	Bos taurus	Blood	12
MH057416.1	Colombia	Bos taurus	Blood	4
MH057417.1	Colombia	Bos taurus	Blood	1
MH057418.1	Colombia	Bos taurus	Blood	4
MH057419.1	Colombia	Bos taurus	Blood	4
MH057420.1	Colombia	Bos taurus	Blood	14
MH057421.1	Colombia	Bos taurus	Blood	4
MH057422.1	Colombia	Bos taurus	Blood	4
MH057423.1	Colombia	Bos taurus	Blood	2
MH057424.1	Colombia	Bos taurus	Blood	1
MH057425.1	Colombia	Bos taurus	Blood	1
MH057426.1	Colombia	Bos taurus	Blood	1
MH057427.1	Colombia	Bos taurus	Blood	1
MH057428.1	Colombia	Bos taurus	Blood	3
MH057429.1	Colombia	Bos taurus	Blood	1
MH057430.1	Colombia	Bos taurus	Blood	4
MH057431.1	Colombia	Bos taurus	Blood	11
MH057432.1	Colombia	Bos taurus	Blood	1
MH057433.1	Colombia	Bos taurus	Blood	11
MH057434.1	Colombia	Bos taurus	Blood	11
MH057435.1	Colombia	Bos taurus	Blood	1
MH057436.1	Colombia	Bos taurus	Blood	1
MH057437.1	Colombia	Bos taurus	Blood	13
MH057438.1	Colombia	Bos taurus	Blood	4
MH057439.1	Colombia	Bos taurus	Blood	4
MH057440.1	Colombia	Bos taurus	Blood	12
MH057441.1	Colombia	Bos taurus	Blood	1
MH057442.1	Colombia	Bos taurus	Blood	1
MH057443.1	Colombia	Bos taurus	Blood	1

Accession Number	Country	Host	Isolation Source	Hap.
MH057444.1	Colombia	Bos taurus	Blood	11
MH057445.1	Colombia	Bos taurus	Blood	1
MH057446.1	Colombia	Bos taurus	Blood	1
MH057447.1	Colombia	Bos taurus	Blood	1
MH057448.1	Colombia	Bos taurus	Blood	1
MH057449.1	Colombia	Bos taurus	Blood	1
MH057450.1	Colombia	Bos taurus	Blood	1
MH057451.1	Colombia	Bos taurus	Blood	1
MH057452.1	Colombia	Bos taurus	Blood	1
MH057453.1	Colombia	Bos taurus	Blood	1
MH057454.1	Colombia	Bos taurus	Blood	1
MH057455.1	Colombia	Bos taurus	Blood	4
MH057456.1	Colombia	Bos taurus	Blood	1
MH057457.1	Colombia	Bos taurus	Blood	1
MH057458.1	Colombia	Bos taurus	Blood	1
MH057459.1	Colombia	Bos taurus	Blood	1
MH057460.1	Colombia	Bos taurus	Blood	1
MH057461.1	Colombia	Bos taurus	Blood	1
MH057462.1	Colombia	Bos taurus	Blood	1
MH057463.1	Colombia	Bos taurus	Blood	1
MH057464.1	Colombia	Bos taurus	Blood	4
MH057465.1	Colombia	Bos taurus	Blood	1
MH170027	Vietnam	Bos taurus	Blood	4
MH170028	Vietnam	Bos taurus	Blood	4
MH170029	Vietnam	Bos taurus	Blood	10
MH170030	Vietnam	Bos taurus	Blood	9
MH293473.1	Colombia	Food product	Beef	1
MH293474.1	Colombia	Food product	Beef	4
MH293475.1	Colombia	Food product	Beef	1
MH293476.1	Colombia	Food product	Beef	1
MH293477.1	Colombia	Food product	Beef	4
MH293478.1	Colombia	Food product	Beef	5
MH293479.1	Colombia	Food product	Beef	4
MH293480.1	Colombia	Food product	Beef	5
MH293481.1	Colombia	Food product	Beef	1
MH293482.1	Colombia	Food product	Beef	1
MH293483.1	Colombia	Food product	Beef	1
MH293484.1	Colombia	Food product	Beef	1
MH293485.1	Colombia	Food product	Beef	4
MH293486.1	Colombia	Food product	Beef	4
MH293487.1	Colombia	Food product	Milk	1
MH293488.1	Colombia	Food product	Milk	1

Accession Number	Country	Host	Isolation Source	Hap.
MH293489.1	Colombia	Food product	Milk	4
MH293490.1	Colombia	Food product	Milk	1
MH293491.1	Colombia	Food product	Milk	1
MH293492.1	Colombia	Food product	Milk	4
MH293493.1	Colombia	Food product	Milk	4
MH293494.1	Colombia	Food product	Milk	4
MH293495.1	Colombia	Food product	Milk	4
MH293496.1	Colombia	Food product	Milk	4
MH293497.1	Colombia	Food product	Milk	4
MH293498.1	Colombia	Food product	Milk	4
MH293499.1	Colombia	Food product	Milk	1
MH293500.1	Colombia	Food product	Milk	4
MH293501.1	Colombia	Food product	Milk	4
NC_001414	RefSeq	NA	Clone	8
MN831962.1	Colombia	Homo Sapiens	Breast	4
MN831961.1	Colombia	Homo Sapiens	Breast	1
MN831960.1	Colombia	Homo Sapiens	Breast	1
MN831959.1	Colombia	Homo Sapiens	Breast	1
MN831958.1	Colombia	Homo Sapiens	Breast	3
MN831957.1	Colombia	Homo Sapiens	Breast	4
MN831956.1	Colombia	Homo Sapiens	Breast	1
MN831955.1	Colombia	Homo Sapiens	Breast	2
MN831954.1	Colombia	Homo Sapiens	Breast	4
MN831953.1	Colombia	Homo sapiens	Blood	4
MN831952.1	Colombia	Homo Sapiens	Breast	2
MN831951.1	Colombia	Homo Sapiens	Breast	4
MN831950.1	Colombia	Homo Sapiens	Breast	4
MN831949.1	Colombia	Homo Sapiens	Breast	4
MN831948.1	Colombia	Homo Sapiens	Breast	1
MN831947.1	Colombia	Homo sapiens	Blood	7
MN831946.1	Colombia	Homo sapiens	Blood	2
MN831945.1	Colombia	Homo Sapiens	Breast	2
MN831944.1	Colombia	Homo sapiens	Blood	2
MN831943.1	Colombia	Homo Sapiens	Breast	2
MN831942.1	Colombia	Homo sapiens	Blood	2

Accession Number	Country	Host	Isolation Source	Hap.
MN831941.1	Colombia	Homo Sapiens	Breast	1
MN831940.1	Colombia	Homo Sapiens	Breast	6
MN831939.1	Colombia	Homo Sapiens	Breast	4
MN831938.1	Colombia	Homo Sapiens	Breast	1
MN831937.1	Colombia	Homo Sapiens	Breast	3
MN831936.1	Colombia	Homo Sapiens	Breast	4
MN831935.1	Colombia	Homo Sapiens	Blood	2
MN831934.1	Colombia	Homo Sapiens	Breast	1
MN831933.1	Colombia	Homo Sapiens	Breast	2
MN831932.1	Colombia	Homo Sapiens	Breast	5
MN831931.1	Colombia	Homo Sapiens	Breast	1
MN831930.1	Colombia	Homo sapiens	Blood	5
MN831929.1	Colombia	Homo Sapiens	Breast	2
MN831928.1	Colombia	Homo Sapiens	Breast	5
MN831927.1	Colombia	Homo Sapiens	Breast	4
MN831926.1	Colombia	Homo Sapiens	Breast	1
MN831925.1	Colombia	Homo Sapiens	Breast	1
MN831924.1	Colombia	Homo Sapiens	Blood	4
MN831923.1	Colombia	Homo Sapiens	Breast	4
MN831922.1	Colombia	Homo Sapiens	Blood	1
MN831921.1	Colombia	Homo Sapiens	Breast	1
MN831920.1	Colombia	Homo Sapiens	Breast	4
MN831919.1	Colombia	Homo Sapiens	Breast	4
MN831918.1	Colombia	Homo Sapiens	Blood	2
MN831917.1	Colombia	Homo Sapiens	Breast	2
MN831916.1	Colombia	Homo Sapiens	Breast	2
MN831915.1	Colombia	Homo Sapiens	Breast	4
MN831914.1	Colombia	Homo Sapiens	Blood	2
MN831913.1	Colombia	Homo Sapiens	Breast	4

Accession Number	Country	Host	Isolation Source	Hap.
MN831912.1	Colombia	Homo Sapiens	Breast	4
MN831911.1	Colombia	Homo Sapiens	Breast	4
MN831910.1	Colombia	Homo Sapiens	Breast	1
MN831909.1	Colombia	Homo Sapiens	Breast	1
MN831908.1	Colombia	Homo Sapiens	Blood	4
MN831907.1	Colombia	Homo Sapiens	Blood	4
MN831906.1	Colombia	Homo Sapiens	Blood	1
MN831905.1	Colombia	Homo Sapiens	Breast	2
MN831904.1	Colombia	Homo Sapiens	Breast	1
MN831903.1	Colombia	Homo sapiens	Blood	1
MN831902.1	Colombia	Homo Sapiens	Breast	1
MN831901.1	Colombia	Homo Sapiens	Breast	3
MN831900.1	Colombia	Homo Sapiens	Breast	2
MN831899.1	Colombia	Homo sapiens	Blood	1
MN831898.1	Colombia	Homo Sapiens	Breast	2
MN831897.1	Colombia	Homo sapiens	Blood	1
MN831896.1	Colombia	Homo sapiens	Blood	1

Table S2. Sequences obtained in the current study from female samples collected from breast and blood. Population description.

Accession	Code	Population	Participant	Age	Type of sample	Isolation Source	Hap.
**MN831896.1**	PUJ_COL_GAG_91HMS	Mederi Hospital	Patient	51	Benign	Blood	1
**MN831897.1**	PUJ_COL_GAG_89HMS	Mederi Hospital	Patient	68	Malignant	Blood	1
**MN831898.1**	PUJ_COL_GAG_88HMT	Mederi Hospital	Patient	70	Malignant	Breast	2
**MN831899.1**	PUJ_COL_GAG_86HMS	Mederi Hospital	Patient	45	Benign	Blood	1
**MN831900.1**	PUJ_COL_GAG_85HMT	Mederi Hospital	Patient	20	Benign	Breast	2
**MN831901.1**	PUJ_COL_GAG_82HMT	Mederi Hospital	Patient	68	Malignant	Breast	3
**MN831902.1**	PUJ_COL_GAG_79ML	INML	Deceased	70	No tumor	Breast	1
**MN831903.1**	PUJ_COL_GAG_76HMS	Mederi Hospital	Patient	75	Malignant	Blood	1

Accession	Code	Population	Participant	Age	Type of sample	Isolation Source	Hap.
**MN831904.1**	PUJ_COL_GAG _73ML	INML	Deceased	58	No tumor	Breast	1
**MN831905.1**	PUJ_COL_GAG _73HMT	Mederi Hospital	Patient	65	Malignant	Breast	2
**MN831906.1**	PUJ_COL_GAG _73HMS	Mederi Hospital	Patient	65	Malignant	Blood	1
**MN831907.1**	PUJ_COL_GAG _71HMS	Mederi Hospital	Patient	62	Benign	Blood	4
**MN831908.1**	PUJ_COL_GAG _70HMS	Mederi Hospital	Patient	70	Malignant	Blood	4
**MN831909.1**	PUJ_COL_GAG _69ML	INML	Deceased	20	No tumor	Breast	1
**MN831910.1**	PUJ_COL_GAG _68ML	INML	Deceased	33	No tumor	Breast	1
**MN831911.1**	PUJ_COL_GAG _67ML	INML	Deceased	78	No tumor	Breast	4
**MN831912.1**	PUJ_COL_GAG _67HMT	Mederi Hospital	Patient	71	Benign	Breast	4
**MN831913.1**	PUJ_COL_GAG _66HMT	Mederi Hospital	Patient	71	Malignant	Breast	4
**MN831914.1**	PUJ_COL_GAG _54HMS	Mederi Hospital	Patient	79	Benign	Blood	2
**MN831915.1**	PUJ_COL_GAG _46HMT	Mederi Hospital	Patient	21	Benign	Breast	4
**MN831916.1**	PUJ_COL_GAG _42HMT	Mederi Hospital	Patient	55	Malignant	Breast	2
**MN831917.1**	PUJ_COL_GAG _39HMT	Mederi Hospital	Patient	73	Malignant	Breast	2
**MN831918.1**	PUJ_COL_GAG _39HMS	Mederi Hospital	Patient	73	Malignant	Blood	2
**MN831919.1**	PUJ_COL_GAG _39HMT	Mederi Hospital	Patient	73	Benign	Breast	4
**MN831920.1**	PUJ_COL_GAG _33HMT	Mederi Hospital	Patient	47	Malignant	Breast	4
**MN831921.1**	PUJ_COL_GAG _27HMT	Mederi Hospital	Patient	69	Malignant	Breast	1
**MN831922.1**	PUJ_COL_GAG _27HMS	Mederi Hospital	Patient	69	Malignant	Blood	1
**MN831923.1**	PUJ_COL_GAG _19HMT	Mederi Hospital	Patient	24	Benign	Breast	4
**MN831924.1**	PUJ_COL_GAG _19HMS	Mederi Hospital	Patient	24	Benign	Blood	4
**MN831925.1**	PUJ_COL_GAG _18ML	INML	Deceased	25	No tumor	Breast	1
**MN831926.1**	PUJ_COL_GAG _15ML	INML	Deceased	52	No tumor	Breast	1
**MN831927.1**	PUJ_COL_GAG _158HMT	Mederi Hospital	Patient	75	Malignant	Breast	4
**MN831928.1**	PUJ_COL_GAG _151HMT	Mederi Hospital	Patient	69	Malignant	Breast	5
**MN831929.1**	PUJ_COL_GAG _150HMT	Mederi Hospital	Patient	80	Malignant	Breast	2

Accession	Code	Population	Participant	Age	Type of sample	Isolation Source	Hap.
**MN831930.1**	PUJ_COL_GAG _150HMS	Mederi Hospital	Patient	80	Malignant	Blood	5
**MN831931.1**	PUJ_COL_GAG _14ML	INML	Deceased	41	No tumor	Breast	1
**MN831932.1**	PUJ_COL_GAG _149HMT	Mederi Hospital	Patient	79	Malignant	Breast	5
**MN831933.1**	PUJ_COL_GAG _144HMT	Mederi Hospital	Patient	51	Malignant	Breast	2
**MN831934.1**	PUJ_COL_GAG _138ML	INML	Deceased	40	No tumor	Breast	1
**MN831935.1**	PUJ_COL_GAG _133HMS	Mederi Hospital	Patient	23	Benign	Blood	2
**MN831936.1**	PUJ_COL_GAG _130HMT	Mederi Hospital	Patient	58	Benign	Breast	4
**MN831937.1**	PUJ_COL_GAG _12HMT	Mederi Hospital	Patient	34	Benign	Breast	3
**MN831938.1**	PUJ_COL_GAG _129ML	INML	Deceased	40	No tumor	Breast	1
**MN831939.1**	PUJ_COL_GAG _126ML	INML	Deceased	89	No tumor	Breast	4
**MN831940.1**	PUJ_COL_GAG _120HMT	Mederi Hospital	Patient	66	Malignant	Breast	6
**MN831941.1**	PUJ_COL_GAG _11ML	INML	Deceased	50	No tumor	Breast	1
**MN831942.1**	PUJ_COL_GAG _119HMS	Mederi Hospital	Patient	50	Malignant	Blood	2
**MN831943.1**	PUJ_COL_GAG _118HMT	Mederi Hospital	Patient	62	Malignant	Breast	2
**MN831944.1**	PUJ_COL_GAG _118HMS	Mederi Hospital	Patient	62	Malignant	Blood	2
**MN831945.1**	PUJ_COL_GAG _117HMT	Mederi Hospital	Patient	25	Benign	Breast	2
**MN831946.1**	PUJ_COL_GAG _116HMS	Mederi Hospital	Patient	63	Benign	Blood	2
**MN831947.1**	PUJ_COL_GAG _115HMS	Mederi Hospital	Patient	50	Benign	Blood	7
**MN831948.1**	PUJ_COL_GAG _113ML	INML	Deceased	76	No tumor	Breast	1
**MN831949.1**	PUJ_COL_GAG _110ML	INML	Deceased	51	No tumor	Tissue	4
**MN831950.1**	PUJ_COL_GAG _110HMT	Mederi Hospital	Patient	29	Benign	Breast	4
**MN831951.1**	PUJ_COL_GAG _109HMT	Mederi Hospital	Patient	27	Benign	Breast	4
**MN831952.1**	PUJ_COL_GAG _107HMT	Mederi Hospital	Patient	52	Pre-malignant	Breast	2
**MN831953.1**	PUJ_COL_GAG _107HMS	Mederi Hospital	Patient	52	Pre-malignant	Blood	4
**MN831954.1**	PUJ_COL_GAG _106HMT	Mederi Hospital	Patient	46	Malignant	Breast	4
**MN831955.1**	PUJ_COL_GAG _104HMT	Mederi Hospital	Patient	70	Malignant	Breast	2

Accession	Code	Population	Participant	Age	Type of sample	Isolation Source	Hap.
**MN831956.1**	PUJ_COL_GAG _09ML	INML	Deceased	63	No tumor	Breast	1
**MN831957.1**	PUJ_COL_GAG _09HMT	Mederi Hospital	Patient	24	Pre-malignant	Breast	4
**MN831958.1**	PUJ_COL_GAG _08HMT	Mederi Hospital	Patient	63	Benign	Breast	3
**MN831959.1**	PUJ_COL_GAG _07ML	INML	Deceased	ND	No tumor	Breast	1
**MN831960.1**	PUJ_COL_GAG _06ML	INML	Deceased	ND	No tumor	Breast	1
**MN831961.1**	PUJ_COL_GAG _05ML	INML	Deceased	65	No tumor	Breast	1
**MN831962.1**	PUJ_COL_GAG _02ML	INML	Deceased	57	No tumor	Breast	4

**INML* – Instituto Nacional de Medicina Legal.

Sequences distribution:

Participants: Patients (n=48), Deceased (n=19)

Type of sample: Malignant (n=26), Pre-malignant (n=3), Benign (n=19), No tumor (n=19)

Table S3. Haplotypes distribution among humans positive to BLV regarding their exposure factors for viral acquisition. Chi-square bivariate anaylisis of exposure factors vs haplotypes.

BLV EXPOSURE FACTORS*	HAPLOTYPES PRESENT IN HUMANS							
	1 n (%)	2 n (%)	3 n (%)	4 n (%)	5 n (%)	6 n (%)	7 n (%)	*P* value
Dairy products and raw milk consumption	14 (26.42)	13 (24.53)	3 (5.66)	17 (32.08)	2 (3.77)	1 (1.89)	1 (1.89)	0.965
Beef consumption	14 (26.42)	13 (24.53)	3 (5.66)	18 (33.96)	2 (3.77)	1 (1.89)	1 (1.89)	0.829
Contact with cattle	4 (9.30)	4 (9.30)	2 (4.65)	6 (13.95)	1 (2.33)	0	0	0.75
Contact with blood	1 (2.44)	3 (7.32)	0	2 (4.88)	2 (4.88)	0	0	0.135
Living in shared environment with cattle	4 (7.55)	3 (5.66)	0	7 (13.21)	2 (3.77)	0	0	0.241

* BLV sequences from humans (n=67). Not all the participants answered the survey of exposure factors. Missing data (n=14).
